# Deep abscopal response to radiotherapy and anti-PD-1 in an oligometastatic melanoma patient with unfavorable pretreatment immune signature

**DOI:** 10.1007/s00262-020-02587-8

**Published:** 2020-04-29

**Authors:** Tsubasa Watanabe, Elke Firat, Jutta Scholber, Simone Gaedicke, Corinne Heinrich, Ren Luo, Nicolas Ehrat, Gabriele Multhoff, Annette Schmitt-Graeff, Anca-Ligia Grosu, Amir Abdollahi, Jessica C. Hassel, Dagmar von Bubnoff, Frank Meiss, Gabriele Niedermann

**Affiliations:** 1 Department of Radiation Oncology, Faculty of Medicine, University of Freiburg, Robert-Koch-Strasse 3, 79106 Freiburg, Germany; 2 Institute for Integrated Radiation and Nuclear Science, Kyoto University, Osaka, Japan; 3 Department of Dermatology and Venerology, Faculty of Medicine, University of Freiburg, Freiburg, Germany; 4 Faculty of Biology, University of Freiburg, Freiburg, Germany; 5 Department of Radiation Oncology, Technical University Munich, Munich, Germany; 6 German Cancer Consortium (DKTK), Partner Site Munich, and German Cancer Research Center, Heidelberg, Germany; 7 Institute of Pathology, Faculty of Medicine, University of Freiburg, Freiburg, Germany; 8 German Cancer Consortium (DKTK), Partner Site Freiburg, and German Cancer Research Center, Heidelberg, Germany; 9 Department of Radiation Oncology, Heidelberg University Medical School, Heidelberg Institute of Radiation Oncology (HIRO), National Center for Radiation Research in Oncology (NCOR), Heidelberg, Germany; 10 German Cancer Consortium (DKTK), Partner Site Heidelberg, and German Cancer Research Center, Heidelberg, Germany; 11 Skin Cancer Center, Department of Dermatology and National Center for Tumor Diseases (NCT), University Hospital Heidelberg, Heidelberg, Germany; 12 Department of Dermatology, Allergy, and Venereology, University of Lübeck, Lübeck, Germany

**Keywords:** Hypofractionated radiotherapy, Immune checkpoint blockade, Abscopal effects, Immune signatures

## Abstract

Radiotherapy can elicit abscopal effects in non-irradiated metastases, particularly under immune checkpoint blockade (ICB). We report on two elderly patients with oligometastatic melanoma treated with anti-PD-1 and stereotactic body radiation therapy (SBRT). Before treatment, patient 1 showed strong tumor infiltration with exhausted CD8+ T cells and high expression of T cell-attracting chemokines. This patient rapidly mounted a complete response, now ongoing for more than 4.5 years. Patient 2 exhibited low CD8+ T cell infiltration and high expression of immunosuppressive arginase. After the first SBRT, his non-irradiated metastases did not regress and new metastases occurred although neoepitope-specific and differentiation antigen-specific CD8+ T cells were detected in the blood. A second SBRT after 10 months on anti-PD-1 induced a radiologic complete response correlating with an increase in activated PD-1-expressing CD8 T cells. Apart from a new lung lesion, which was also irradiated, this deep abscopal response lasted for more than 2.5 years. However, thereafter, his disease progressed and the activated PD-1-expressing CD8 T cells dropped. Our data suggest that oligometastatic patients, where a large proportion of the tumor mass can be irradiated, are good candidates to improve ICB responses by RT, even in the case of an unfavorable pretreatment immune signature, after progression on anti-PD-1, and despite advanced age. Besides repeated irradiation, T cell epitope-based immunotherapies (e.g., vaccination) may prolong antitumor responses even in patients with unfavorable pretreatment immune signature.

**Electronic supplementary material:**

The online version of this article (10.1007/s00262-020-02587-8) contains supplementary material, which is available to authorized users.

## Background

The standard of care for metastatic melanoma is immune checkpoint blockade (ICB) or targeted therapy [1]. In the absence of brain metastases, pembrolizumab and nivolumab monotherapy yield durable responses in approx. 30–40% of patients; the complete response (CR) rate is approx. 15% [2, 3]. Additional radiotherapy (RT) may increase the rate of deep and durable responses. Preclinical work has shown that localized RT can induce CD8+ T cells, which contribute to the control of the irradiated tumor and sometimes elicit abscopal effects in non-irradiated metastases, particularly when combined with ICB [4–7]. Case reports [8] and clinical trials have also provided evidence for RT-induced abscopal effects [9–14]. However, it is not fully clear how to best induce an RT-mediated abscopal response and whether pretreatment biomarkers can predict which patients respond to combined RT/ICB.

Melanoma has been regarded as radioresistant, and, in melanoma, RT is currently mainly used in the adjuvant setting or to palliate symptoms [15]. Advanced age is usually a disadvantage for immunotherapy, mainly because of immunosenescence, but anecdotal clinical experience suggests that advanced age does not result in poorer responses in elderly patients treated with ICB [16].

Here, we report on two elderly patients with oligometastatic melanoma who mounted a deep (long-lasting complete or radiologic complete) response to stereotactic body radiotherapy (SBRT) and anti-PD-1. Patient 1 had a favorable pretreatment immune signature and immediately responded with a CR, now already lasting for more than 4.5 years. Patient 2 had an unfavorable pretreatment immune signature. Nonetheless, he showed a radiologic CR after a second (late) SBRT delivered after more than 10 months of progression on anti-PD-1 and the first SBRT. However, eventually, his disease progressed. Besides various potential pre- and on-treatment biomarkers, we also characterized CD8+ T cell responses to melanoma differentiation antigens and neoantigens. The latter could be useful for the design of additional immunotherapies that might further deepen responses, including patients with unfavorable immune signature.

## Materials and methods

### Patient study

All human samples were collected after approval by the Ethics Committee of the Albert-Ludwigs University Freiburg, Germany (protocol no. 453/14) following written informed consent.

### Whole-exome sequencing, RNA sequencing, HLA typing, and neoepitope prediction

DNA and RNA were extracted from formalin-fixed, paraffin-embedded tumor sections and PBMCs. Sequencing was performed by Personalis (Menlo Park, USA), and gene expression profiles and potential neoepitope lists were generated. HLA typing was also performed by next-generation sequencing. The neoepitopes were chosen by the rank of the predicted affinity using NetMHCpan 4.0, the ratio of wild-type- and tumor-binding rank, and based on source protein expression. Transcripts per million base pairs were used to compare gene expression levels between the two patients.

### Immunohistochemistry (IHC)

Tumor sections were stained with hematoxylin and eosin (H&E) or with primary antibodies to CD8 (clone C8/144B from Dako-Agilent) and PD-L1 (Ventana clone SP142 from Roche). IHC staining was conducted using K8020 Envision Flex (for brown stainings) or K5005 alkaline phosphatase detection kits (for red stainings) in a DAKO Plus Austostainer.

### Detection of differentiation antigen- and neoepitope-specific CD8+ T cells

PBMCs were incubated with 13 potential neoepitope peptides or HLA-A2-restricted peptides derived from differentiation antigens (each 10 μg/ml) plus anti-CD28 (0.5 μg/ml) (CD28.2). The following differentiation antigen-derived epitopes were used: gp100_209–217_ (IMDQVPFSV), MART-1_26–35_ (ELAGIGILTV), gp100_280–288_ (YLEPGPVTV), and tyrosinase_369–377_ (YMDGTMSQV). From day 2 onwards, IL-2 (20–100 IU/ml) was added. Every 14 days, the cultures were restimulated with peptide-pulsed, 40-Gy-irradiated autologous PBMCs. Epitope-specific T cell assays were performed after a 6-h restimulation with the respective peptide pools or with individual peptides. After 1 h, brefeldin A was added. Cells were then stained for surface CD8+ and intracellular IFNγ and analyzed by flow cytometry.

### Flow cytometry

Cells were stained with Zombie Red and then with antibodies against CD3 (OK3), CD8 (SK1), PD-1 (EH12.2H7), CD45RA (HI100), and CCR7 (G043H7). For nuclear staining of Ki67 (Ki67), we used the eBioscience Fixation/Permeabilisation kit. For IFNγ (clone 4S.B3) staining, the Fixation and Permeabilization buffer from Invitrogen was used. Cells were analyzed on a CytoflexS cytometer (Beckman Coulter).

## Results

### Case history

**Fig. 1:**
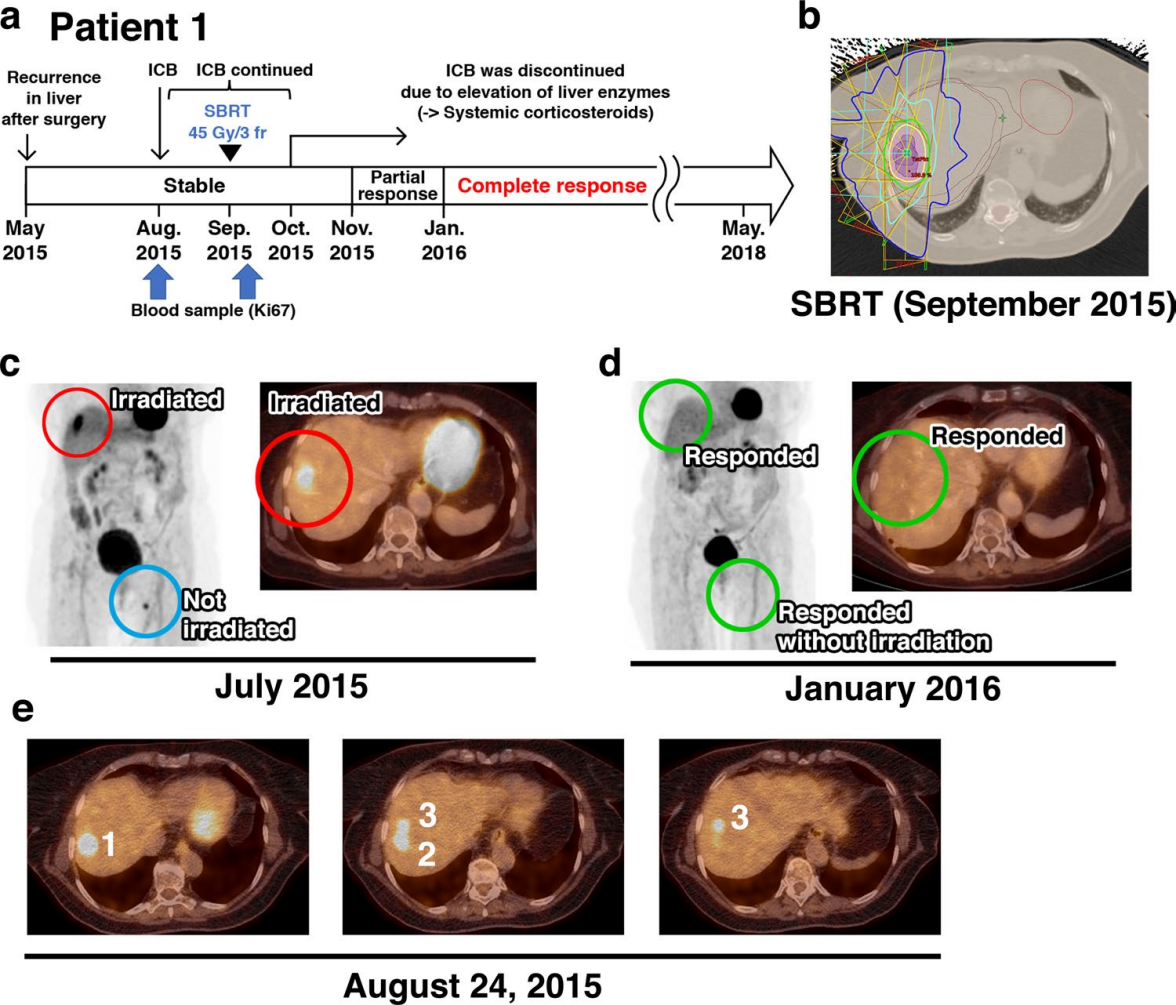
**a** Disease and treatment course for patient 1. **b** Irradiation field. SBRT in September 2015 was delivered in 3 fractions of 15 Gy on consecutive days to three liver lesions located close together. **c**, **d** FDG-PET/CT before (**c**) and 5 months after the ICB initiation/4 months after SBRT (**d**). Irradiated lesions are highlighted by red circles, non-irradiated lesions by blue circles, and responding lesions by green circles. **e** FDG-PET/CT sections showing the three closely spaced liver lesions (numbered 1–3) that were irradiated. *ICB* Immune checkpoint blockade, *SBRT* stereotactic body radiation therapy

**Case** **1** In January 2012, a 69-year-old woman was diagnosed with *BRAF*-wild-type melanoma on the upper left leg. The primary tumor and the sentinel inguinal lymph nodes were resected. The disease was staged pT2a pN1a cM0. From 5/2012 to 12/2012, the patient received low-dose adjuvant IFNα therapy. In May 2015, she presented with macroscopic lymph node metastases in the left groin, which were R1-resected (Fig. 1a). In June 2015, three liver metastases, a muscle lesion in the upper left leg, and a lesion in the left groin were detected by FDG-PET/CT. Since only three body regions were affected, with a limited number of macroscopic metastases, the patient was treated with potentially curative SBRT to the three liver lesions (Fig. 1b, c, e). The SBRT (3 fractions of 15 Gy on consecutive days) was delivered in September 2015, 4 weeks after initiation of nivolumab (between the 3rd and 4th nivolumab doses) (Fig. 1a). Due to non-symptomatic grade 3 elevation of liver enzymes, anti-PD-1 was discontinued after the 6th nivolumab infusion, and systemic corticosteroids were initiated. The first response evaluation (12 weeks after the nivolumab initiation/8 weeks after SBRT) showed a partial response of all irradiated and non-irradiated lesions. At 6 months after the nivolumab initiation/5 months after SBRT, FDG-PET/CT showed CR, despite early nivolumab discontinuation and continued corticosteroids (Fig. 1d). In March 2020, more than 4.5 years after the concurrent SBRT and nivolumab, there was still no evidence of disease.

**Fig. 2:**
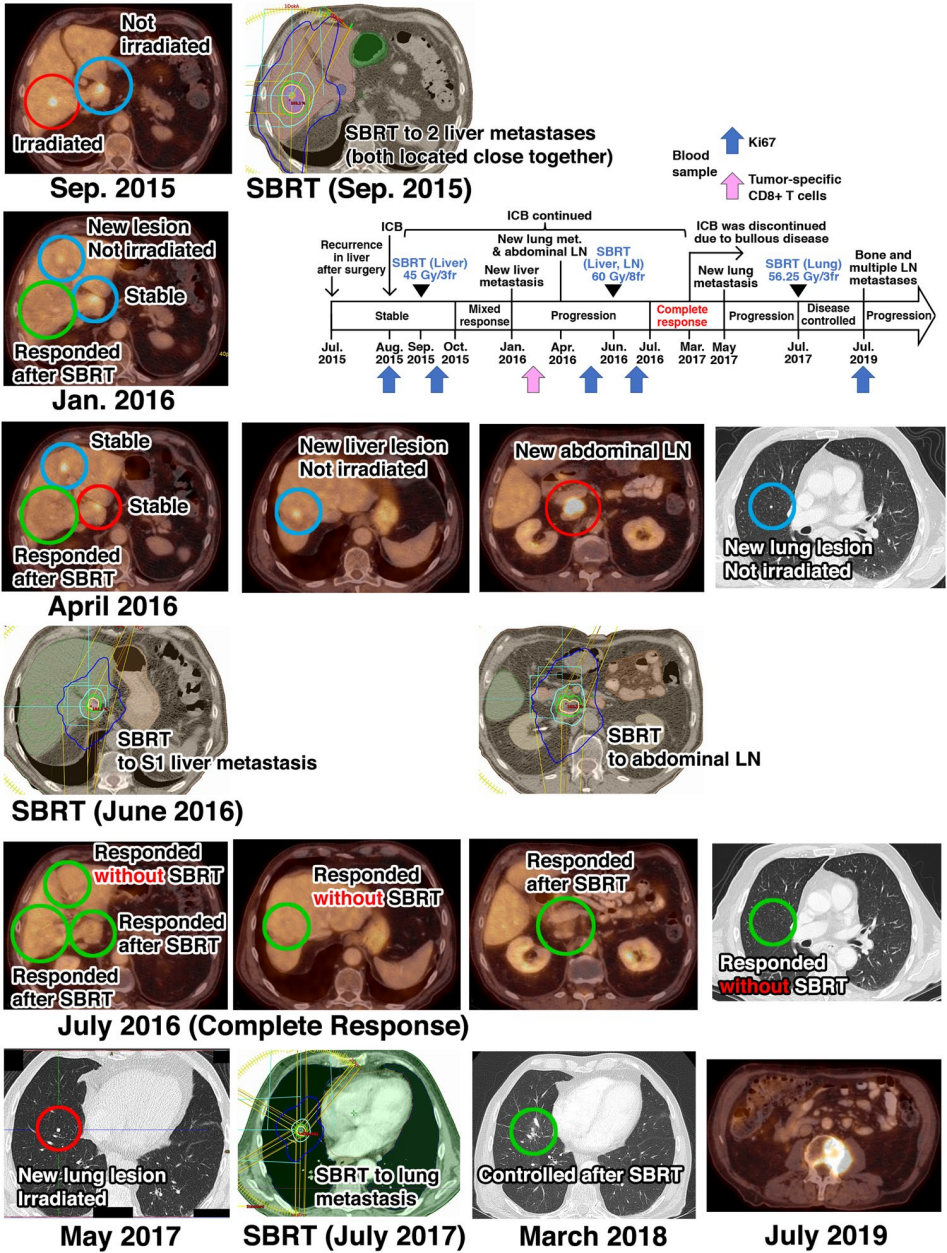
Disease and treatment course for patient 2 as well as FDG-PET/CTs and irradiation plans. The 1st SBRT in September 2015 was delivered in 3 fractions of 15 Gy every other day to the two largest lesions located close together in a non-central liver region. The 2nd SBRT in June 2016 was delivered in 8 fractions of 7.5 Gy over 2.5 weeks to the abdominal lymph node and a liver metastasis. The 3rd SBRT in July 2017 was delivered in 3 fractions of 18.75 Gy every other day to the new lung lesion. Irradiated lesions are highlighted by red circles, non-irradiated lesions by blue circles, and responding lesions by green circles. *ICB* Immune checkpoint blockade, *SBRT* stereotactic body radiation therapy, *LN* lymph node

**Case** **2** In September 2013, a 77-year-old man was diagnosed with *BRAF*-wild-type melanoma in the right breast region; the primary tumor and the sentinel lymph nodes in the axilla were resected. The disease was staged pT3b pN1a cM0. The patient refused adjuvant IFNα therapy. In February 2015, a solitary liver metastasis was detected, which was surgically resected. In July 2015, CT revealed three new liver metastases. Since only one organ showed a limited number of macroscopic metastases, potentially curative SBRT (3 fractions of 15 Gy every other day) was delivered to the two largest lesions located close together in a non-central liver region. The SBRT was delivered in September 2015, 4 weeks after the pembrolizumab initiation (between the 2nd and 3rd pembrolizumab doses) (Fig. 2). At the first response evaluation (10 weeks after the pembrolizumab initiation/6 weeks after SBRT), the irradiated lesions had regressed and the non-irradiated lesion was stable. At 5 months after the pembrolizumab initiation/4 months after SBRT, FDG-PET/CT revealed progression, with a new liver lesion. Three months later (April 2016), a new lung lesion and another new liver lesion appeared; in addition, a large abdominal lymph node metastasis was detected (Fig. 2). The tumor board decided to again try to induce an abscopal effect by delivering a second SBRT (8 fractions of 7.5 Gy over 2.5 weeks) to the large lymph node metastasis and one liver metastasis; the three other detectable lesions (two liver lesions and the lung lesion) were not irradiated. The 2nd SBRT was delivered in June 2016, after the 14th cycle of pembrolizumab (Fig. 2). FDG-PET/CT in July 2016 revealed a CR with radiologic complete regression of all irradiated and non-irradiated metastases, i.e., a strong RT-induced abscopal effect (Fig. 2).

In September 2016, a pruriginous exanthema appeared which initially responded to topical steroids. In December 2016, the skin symptoms exacerbated and histological, clinical, and blood (BP180-ELISA) examinations led to the diagnosis of autoimmune bullous pemphigoid, likely related to pembrolizumab. Systemic corticosteroids were then administered for approx. 12 months, which improved the skin condition. Pembrolizumab was nevertheless discontinued in March 2017. In May 2017, FDG-PET/CT revealed a new lung lesion, which was treated by SBRT (Fig. 2). Afterward, all lesions were controlled at least until December 2018. However, FDG-PET/CT in July 2019 revealed progression with a new vertebral lesion (Fig. 2), several lymph node metastases in the cervical region, and a suspicious soft-tissue lesion in the left thigh.

After multifocal progression was diagnosed in July 2019, the patient at first refused further therapy apart from RT of the symptomatic vertebral bone metastasis. According to the patient’s will, RT of the 3rd lumbar vertebra metastasis was performed with 5 fractions of 3 Gy per week for 2 weeks (cumulative dose 30 Gy), which led to local tumor control as documented by CT scans in December 2019. But CT scans in December 2019 revealed a further progression with peritoneal tumor spread, and pembrolizumab therapy was resumed in January 2020. Pembrolizumab therapy led to a decrease in the tumor marker S100 and the patient’s general condition is now stable without symptoms of metastatic disease. The bullous pemphigoid has not yet recurred since the reinitiation of pembrolizumab.

### Pretreatment immune signature

**Fig. 3:**
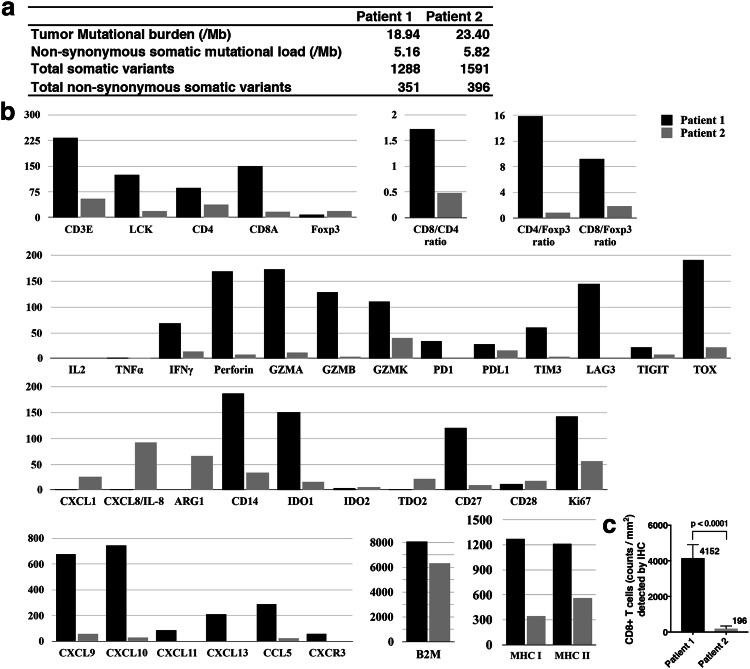
Comparative analysis of the pretreatment tumor immune signatures between patient 1 and patient 2. **a** Tumor mutational burden. **b** RNA expression of various proteins playing a role in T cell signaling, differentiation, exhaustion, and cytotoxicity. Expression levels of chemokines, immunosuppressive enzymes, and β2M as well as of MHC class I and MHC class II were also analyzed. Transcripts per million base pairs were used to compare gene expression levels between the two patients. **c** Density of CD8+ TILs as determined by IHC in pretreatment tumor tissue. *Mb* Megabase

Pretreatment tumor material obtained through surgical resection a few weeks before the start of ICB was analyzed by bulk whole-exome sequencing (Fig. 3a, b, Supplementary Fig. 1) and IHC (Fig. 3c, Supplementary Fig. 2). Both patients’ tumors showed a similar mutational burden (Fig. 3a) in the upper-intermediate range for melanoma [17]. However, there were strong differences in the immune status of the resected tumor material. High levels of CD8+ T cells were found in the resected lymph node metastasis of patient 1; the resected liver metastasis of patient 2 showed a 20 times lower CD8+ T cell density both by RNAseq and by IHC (Fig. 3b, c, Supplementary Fig. 2).

Moreover, the lymph node metastasis of patient 1 revealed an exhaustion signature (Fig. 3b) [18]. Besides PD-1 and PD-L1, the exhaustion markers TIM-3 and LAG-3 and the transcription factor TOX, which is required for the formation of exhausted T cells [19], were expressed. Exhausted T cells secrete high levels of cytotoxic molecules (perforin, granzymes) and the T cell effector cytokine IFNγ, but not IL-2. TNFα expression is also usually reduced. Another typical feature is high expression of IFNγ-inducible T cell-recruiting chemokines (CXCL9, CXCL10), which were found, in addition to the T cell-recruiting chemokine CCL5, and CXCL13 recently reported to be expressed in tertiary lymphoid tissue [20]. Expression of indoleamine 2,3-dioxygenase (IDO1), also typically induced by IFNγ, was also found (Fig. 3b). Remarkably, there was only little evidence for the presence of immunosuppressive leukocytes such as CD4+ Tregs; immunosuppressive arginase typically expressed by immunosuppressive myeloid cells was not found at all.

In contrast, the resected liver metastasis of patient 2 showed no expression of PD-1, TIM-3, or LAG-3. However, some expression of the PD-1 ligand, PD-L1, which can occur dependently or independently of IFNγ [21], was detected (Fig. 3b, Supplementary Fig. 2). Perforin, IFNγ, IL-2, TNFα, and granzymes were virtually undetectable (Fig. 3b). In accordance with the low IFNγ expression, expression of IDO1, CXCL9, and CXCL10 was also low. However, the Treg-specific transcription factor FoxP3 was expressed at higher levels than in the lymph node metastasis of patient 1, and expression of immunosuppressive arginase was also relatively high (Fig. 3b). Expression of TOX was low and CXCL13 was undetectable. Together, these findings did not show evidence for pretreatment tumor infiltration by exhausted T cells that could respond to anti-PD-1.

Both patients’ tumors showed high expression of β2-microglobulin (β2M) as well as expression of MHCI. Expression of MHC class II, typically found on professional antigen presenting cells, was higher in the lymph node metastasis of patient 1 (Fig. 3b).

### Immune pharmacodynamic analyses of PBMCs

**Fig. 4:**
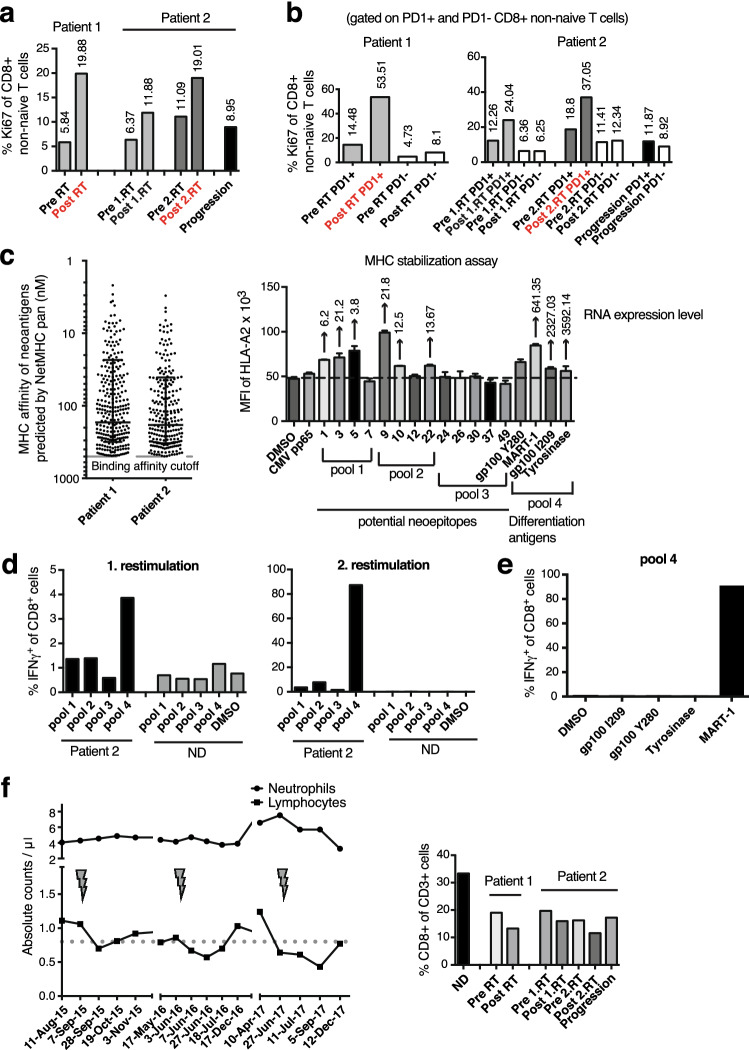
Pharmacodynamic immune response to anti-PD-1 and SBRT. **a** Ki67 in blood CD8 T cells pre and post combined RT/ICB. **b** Ki67 in PD-1+ versus PD-1− CD8 T cells pre and post combined RT/ICB. **c** Predicted MHC I-binding affinity of mutated peptides (left); (right) HLA-A2.1-binding affinity of the selected 13 potential neoepitope peptides (pools 1–3) and four known epitopes derived from expressed melanoma differentiation antigens (pool 4), as determined by T2 assay. In the assay, 10 μg/ml of each peptide and 3 μg/ml β2M were incubated with HLA-A2.1+ T2 cells for 18 h at 37 °C. Relative RNA expression of the source proteins is indicated on top of the bars. **d** Percentage of tumor epitope-specific CD8+ T cells in PBMCs of patient 2 (from January 2016) based on intracellular IFNγ-staining after one and two rounds of restimulation on peptide-pulsed autologous PBMCs. PBMCs from a healthy normal HLA-A2.1 + donor were used as control. **e** The CD8+ T cell responses in patient 2 were mainly directed toward MART-1_26–35_/HLA-A2.1 complexes. **f** Development of lymphopenia during treatment in patient 2 (left). The three courses of SBRT are indicated by arrows. Proportion of CD8+ T cells among CD3 T cells in PBMCs for both patients and a normal donor for control (right). *ND* Normal donor

ICB can induce the proliferation of PD-1+ T cells [22, 23]; it is unclear whether conventional treatments (including different modes of RT) also increase it. We analyzed PBMCs before ICB and 7–10 days after the first SBRT. PBMCs of patient 2 were also analyzed before and after the second SBRT. After initiation of ICB and the subsequent first SBRT, patient 1, who rapidly developed a CR, showed a stronger increase in Ki67+ CD8+ T cells compared to the pretreatment levels (approx. 3.5-fold; post-treatment: 20%) than patient 2 (approx. twofold; post-treatment: 12%) (Fig. 4a). The increase in Ki67 was mainly seen in PD-1+ CD8+ T cells (patient 1: post-treatment 54%; patient 2: 24%) (Fig. 4b). The frequencies of Ki67+ cells indeed seemed to correlate with the treatment response. After the second SBRT in patient 2, which provoked the strong abscopal response despite resistance to long-term anti-PD-1 therapy, the Ki67+ CD8+ T cells increased to 19%, and the PD-1+ CD8+ T cells expressing Ki67, to 37%, i.e., to a similar level as seen in patient 1 after successful combined SBRT/ICB. When patient 2 clearly progressed (August 2019), the CD8+ T cells expressing Ki67 dropped to 9%.

We then investigated whether tumor-specific CD8+ T cells were detectable in the blood of patient 2 during progression between the first and second SBRT. Known HLA-A2-restricted epitopes from melanoma differentiation antigens, which were highly expressed in the pretreatment melanoma tissue, and potential neoepitopes, predicted based on whole-exome DNA and RNA sequencing, were selected (Fig. 4c left). Only a few of these potential neoepitope peptides bound with high affinity to HLA-A2.1 (Fig. 4c right). By using peptide pools, we detected reactivity to differentiation antigens and to neoepitopes (Fig. 4d). The response of differentiation antigen-specific CD8+ T cells, which mainly recognized the MART-1_26–35_ epitope, was particularly high (Fig. 4e). Tumor-specific T cells could be detected despite a pronounced lymphopenia, which deepened with each round of SBRT (Fig. 4f left, Supplementary Fig. 3). Of note, both patients also had an unusually low proportion of CD8+ T cells among the CD3+ cells in the blood (normally approx. 30%) (Fig. 4f right).

The ratio of Ki67+ CD8+ T cells to the tumor burden might be a better predictor for ICB response than Ki67+ T cells alone [23]. In our study, a large proportion of the visible tumor mass was irradiated in the two patients who were “oligometastatic” [24]. In the successful second SBRT in patient 2, more than 90% of the tumor mass was irradiated, thus leaving only a very small non-irradiated tumor mass (approx. 10 cm^3^) (Table 1).

**Table 1:** Irradiated (RT), non-irradiated (abscopal), and total tumor load as detected by CT before each SBRT for both patients

Tumor lesions (cm)^3^	Patient 1	Patient 2		
	Aug. 2015	Sep. 2015	Apr. 2016	May 2017
Liver 1	23.60 (RT)	7.56 (RT)	–	–
Liver 2	4.18 (RT)	10.06 (RT)	–	–
Liver 3	–	8.33	16.6 (RT)	–
Inguinal LN (R1 resection)	Not measurable	–	–	–
Muscle lesion	6.73	–	–	–
Liver 4	–	–	2.93	–
Liver 5	–	–	6.67	–
Abdominal LN	–	–	107.29 (RT)	–
Lung 1	–	–	0.28	–
Lung 2	–	–	–	0.63 (RT)
Total tumor volume	34.51	25.95	133.77	0.63
Irradiated volume	27.78 (80.5%)	17.62 (67.9%)	123.89 (92.6%)	0.63 (100%)

## Discussion

Clinical attempts to induce abscopal effects in metastatic patients have so far been based on the irradiation of one or two tumor nodules, with limited success. Our data suggest that patients with limited or oligometastatic disease, where a large proportion of the tumor mass can be irradiated, are good candidates to increase ICB response rates by RT, even in case of an unfavorable pretreatment immune signature, after progression on long-term anti-PD-1, and despite advanced age. We also show that deep abscopal effects can be achieved through a repeated irradiation, but long-term outcomes may be worse in patients with unfavorable immune signature. However, even in the patient with the unfavorable pretreatment immune signature, tumor epitope-specific T cells could be detected in the blood after the first (ineffective) attempt to induce an abscopal effect by RT, and this could be the basis for additional epitope-based immunotherapies.

Patient 1 had favorable pretreatment biomarkers. However, in sum, her tumor lesions showed a diameter of less than 5 cm and PD-L1 positivity, and therefore she had an approx. 40% chance of achieving a CR upon anti-PD-1 monotherapy [25]. It is therefore unclear if the SBRT contributed to the CR in this patient. In contrast, patient 2 showed a clear RT-induced abscopal response after a second SBRT following progression on long-term anti-PD-1 and the first SBRT. This strong abscopal response occurred despite low CD8+ T cell infiltration, the absence of a T cell exhaustion and cytotoxicity signature, and the presence of immunosuppressive cells in the pretreatment tumor tissue.

Preclinical experiments suggest that non-ablative hypofractionated RT may work best to induce abscopal responses [5, 6]. In our study, both patients initially received ablative SBRT, resulting in CR in patient 1 but no systemic regression in patient 2. The second (effective) SBRT in patient 2 had a curative total dose but non-ablative fraction doses. Moreover, based on the assumption that TILs are radiosensitive, it is usually assumed that repetitive tumor irradiations may be detrimental. However, the systemically effective (second) SBRT in patient 2 consisted of 8 fractions distributed over 2.5 weeks. This is consistent with our recent preclinical work showing that such extended hypofractionated RT schedules are not necessarily less effective than short schedules [26]. It is also consistent with a recent report showing that tumor-resident T cells are quite radioresistant [27].

RT-mediated abscopal effects depend on tumor-specific CD8+ T cells. Therefore, the direct RT-mediated reduction in the tumor mass (which is feasible in limited or oligometastatic disease [24]) may be beneficial, because it likely facilitates T cell-mediated tumor rejection. In our patients, a relatively high proportion of the visible tumor mass (up to > 90%) was irradiated.

In accordance with the poor pretreatment immune signature, the radiologic CR in patient 2 was only transient. However, upon initiation of ICB and the first SBRT (August/September 2015), patient 2 showed an increase in Ki67+ (i.e., activated) CD8+ T cells and of Ki67+ PD-1+ CD8+ T cells in the blood, which was still detected several months later (May 2016) despite tumor progression. In accordance with these findings, differentiation antigen- and neoepitope-specific CD8+ T cells could clearly be detected during this period of post-treatment tumor progression (January 2016). The detection of tumor epitope-specific T cells could be the basis for additional, epitope-based immunotherapies (vaccination, adoptive T cell transfer) that could help to improve antitumor responses.

Easily detectable on-treatment changes in biomarkers are also of interest to identify effective treatment combinations. At the time point of successful systemic/abscopal response, both patients had high levels (approx. 40–50%) of Ki67+ (i.e., proliferating) PD-1+ CD8+ T cells; in patient 2, this increase was induced by RT despite resistance to anti-PD-1. Since the patient was still under PD-1 ICB, it is unclear whether (but possible that) the increase in Ki67+ T cells was induced by the tumor irradiation alone.

In both patients, a strong antitumor response was observed despite early ICB discontinuation and early commencement of systemic corticosteroids after CR. Whereas the bullous disease in patient 2 was clearly related to anti-PD-1 [28], it is unclear to which extent anti-PD-1 and liver SBRT contributed to the transient grade 3 transaminitis in patient 1 [29]. Our data also show that combined SBRT/ICB can be highly effective in elderly patients [30].

Future clinical trials could investigate to which extent (early or late) SBRT enhances ICB response rates in patients with oligometastatic disease where all or large parts of the visible tumor mass can be irradiated. The effects of repeated irradiations could also be addressed in such trials. Our study furthermore suggests that patients with poor pretreatment CD8+ T cell infiltration should not be excluded from attempts to induce RT-mediated systemic/abscopal effects, at least in oligometastatic disease where all or large parts of the visible tumor mass can be irradiated. In addition, our data support the notion that old patients should not be excluded from attempts to induce RT-mediated abscopal effects.

### Electronic supplementary material

Below is the link to the electronic supplementary material.

**Suppl. Figure** **1.** Mutation signature plots from DNA extracted from pretreatment tumor tissue (lymph node metastasis of patient 1 and liver metastasis of patient 2). Most mutations were C > T changes, compatible with UV-induced damage (PDF 932 kb)

**Suppl. Figure** **2.** IHC analyses of pretreatment tumor samples (lymph node metastasis of patient 1 and liver metastasis of patient 2). **a**, H&E staining. **b**, Staining of CD8+ T cells. **c**, PD-L1 staining (PDF 16101 kb)

**Suppl. Figure** **3.** Neutrophil-to-lymphocyte ratio in peripheral blood during the treatment course (PDF 506 kb)
