# Supplementary figures and images for: Deep abscopal response to radiotherapy and anti-PD-1 in an oligometastatic melanoma patient with unfavorable pretreatment immune signature
Source: Cancer Immunol Immunother. 2020 Apr 29;69(9):1823–32. doi: 10.1007/s00262-020-02587-8 (PMC7413872; doi:10.1007/s00262-020-02587-8)

Supplementary Fig. 1

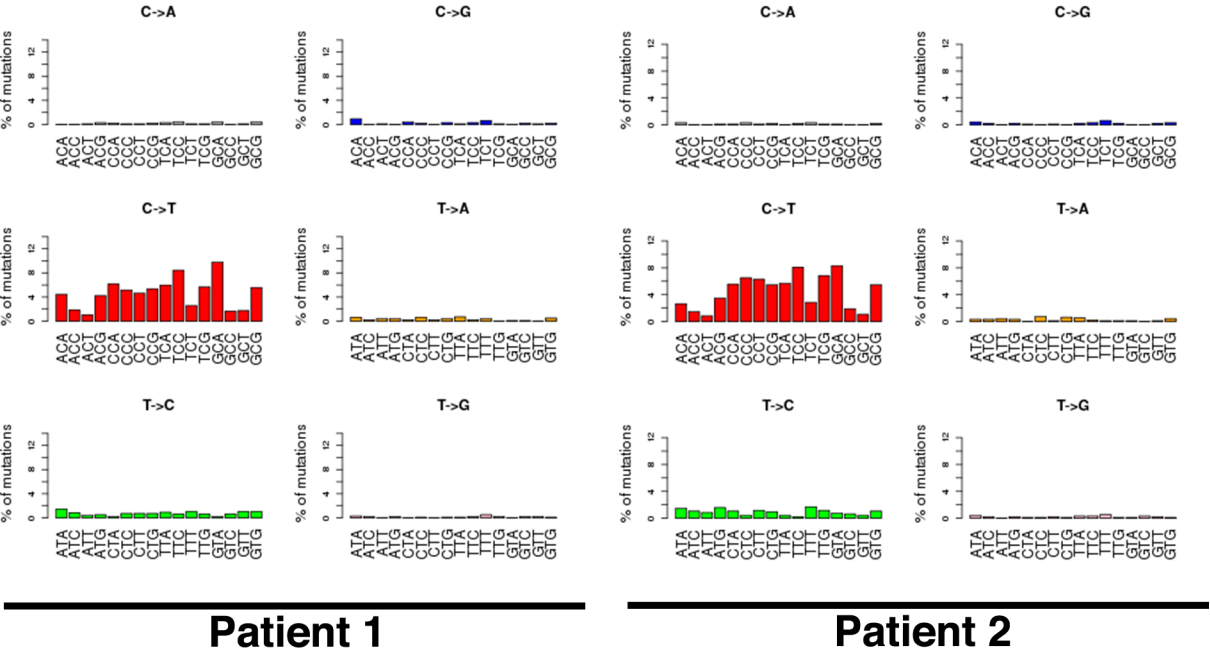

Supplement: Supplementary file 1 — Suppl. Figure 1. Mutation signature plots from DNA extracted from pretreatment tumor tissue (lymph node metastasis of patient 1 and liver metastasis of patient 2). Most mutations were C > T changes, compatible with UV-induced damage (PDF 932 kb) [file 262_2020_2587_MOESM1_ESM.pdf]

Supplementary Fig. 3

**a Patient 1**

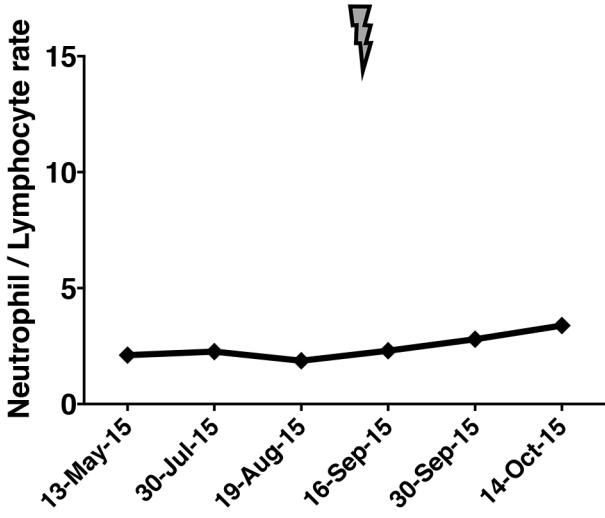

**b Patient 2**

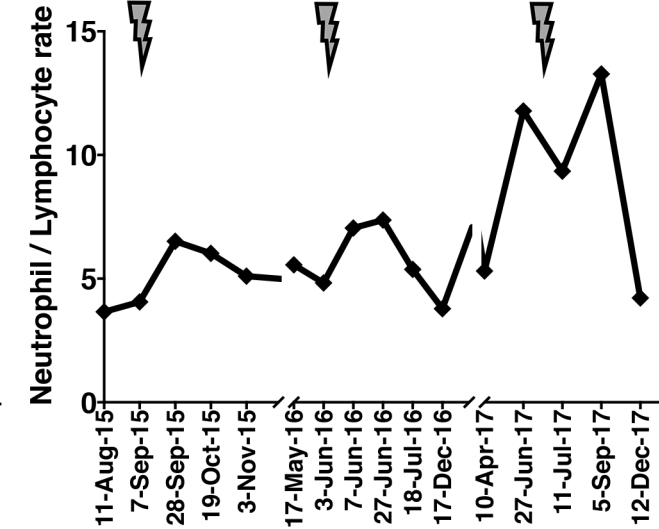

Supplement: Supplementary file 3 — Suppl. Figure 3. Neutrophil-to-lymphocyte ratio in peripheral blood during the treatment course (PDF 506 kb) [file 262_2020_2587_MOESM3_ESM.pdf]
