# Supplementary material for: Deep abscopal response to radiotherapy and anti-PD-1 in an oligometastatic melanoma patient with unfavorable pretreatment immune signature
Source: Cancer Immunol Immunother. 2020 Apr 29;69(9):1823–32. doi: 10.1007/s00262-020-02587-8 (PMC7413872; doi:10.1007/s00262-020-02587-8)

Supplementary Fig. 2

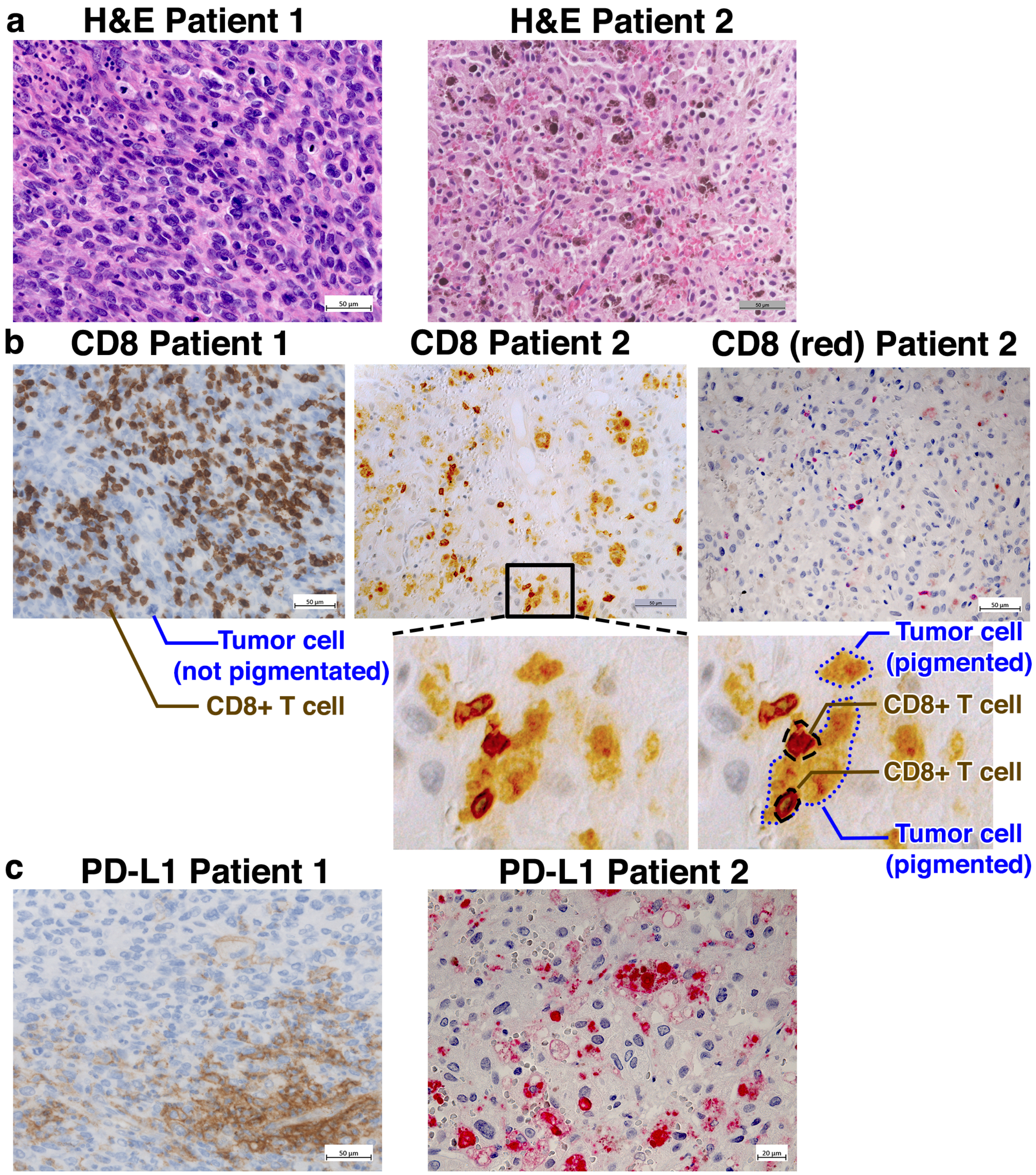

Supplement: Supplementary file 2 — Suppl. Figure 2. IHC analyses of pretreatment tumor samples (lymph node metastasis of patient 1 and liver metastasis of patient 2). a, H&E staining. b, Staining of CD8+ T cells. c, PD-L1 staining (PDF 16101 kb) [file 262_2020_2587_MOESM2_ESM.pdf]
